# Recent Advances in Genome Editing Using CRISPR/Cas9

**DOI:** 10.3389/fpls.2016.00703

**Published:** 2016-05-24

**Authors:** Yuduan Ding, Hong Li, Ling-Ling Chen, Kabin Xie

**Affiliations:** ^1^National Key Laboratory of Crop Genetic Improvement, Huazhong Agricultural UniversityWuhan, China; ^2^College of Informatics, Huazhong Agricultural UniversityWuhan, China; ^3^College of Plant Science and Technology, Huazhong Agricultural UniversityWuhan, China

**Keywords:** CRISPR/Cas9, plants, genome editing, guide RNA, bioinformatic tools

## Abstract

The CRISPR (clustered regularly interspaced short palindromic repeat)-Cas9 (CRISPR-associated nuclease 9) system is a versatile tool for genome engineering that uses a guide RNA (gRNA) to target Cas9 to a specific sequence. This simple RNA-guided genome-editing technology has become a revolutionary tool in biology and has many innovative applications in different fields. In this review, we briefly introduce the Cas9-mediated genome-editing method, summarize the recent advances in CRISPR/Cas9 technology, and discuss their implications for plant research. To date, targeted gene knockout using the Cas9/gRNA system has been established in many plant species, and the targeting efficiency and capacity of Cas9 has been improved by optimizing its expression and that of its gRNA. The CRISPR/Cas9 system can also be used for sequence-specific mutagenesis/integration and transcriptional control of target genes. We also discuss off-target effects and the constraint that the protospacer-adjacent motif (PAM) puts on CRISPR/Cas9 genome engineering. To address these problems, a number of bioinformatic tools are available to help design specific gRNAs, and new Cas9 variants and orthologs with high fidelity and alternative PAM specificities have been engineered. Owing to these recent efforts, the CRISPR/Cas9 system is becoming a revolutionary and flexible tool for genome engineering. Adoption of the CRISPR/Cas9 technology in plant research would enable the investigation of plant biology at an unprecedented depth and create innovative applications in precise crop breeding.

## Introduction

DNA sequencing technology has enabled us to decipher the sequence of whole genomes and to profile the transcriptomes of many organisms with unprecedented throughput, scalability, speed, and low cost. The knowledge of plant genomes and transcriptomes is increasing every day, opening the possibility of creating new crop cultivars through molecular design and genetic engineering. However, our ability to precisely modify plant genomes used to be limited until the emergence of efficient genome-editing technology. This technology uses designer nucleases and the cellular DNA repair system to precisely modify genomic sequence (Voytas, 2013). In practice, a synthetic sequence-specific nuclease is designed to recognize the chosen genomic site and is transfected into the cell, where it creates a double-strand DNA break (DSB) at the site. This DSB is usually repaired by the endogenous, error-prone non-homologous end joining (NHEJ) pathway, which introduces a small insertion or deletion (InDel) at the site, thus knocking out the gene. If a donor DNA fragment with homology to the flanking sequence is present, as with gene knock-in experiments, the DSB is repaired through homology-directed repair (HDR) using the donor DNA as a template. As a result, the sequence of the donor fragment is integrated into the genome at the DSB site. The DSB repair system (NHEJ and HDR) is a ubiquitous component of all living cells; therefore, an artificial nuclease whose recognition site is reprogrammable is the most critical part of genome editing.

To date, three programmable nucleases have been developed for genome editing, including zinc finger nucleases (ZFNs; Pabo et al., 2001), transcription activator–like effector nucleases (TALENs; Boch et al., 2009; Moscou and Bogdanove, 2009), and RNA-guided nucleases (RGNs) from the clustered regularly interspaced short palindromic repeat (CRISPR) and CRISPR-associated proteins (Cas) system (van der Oost, 2013). Among these nucleases, the Cas9 nuclease, which recognizes target DNA according to Watson-Crick base pairing between its guide RNA(s) and DNA, is the simplest one to implement and quickly became the most popular and powerful tool for genome engineering. The advanced CRISPR/Cas9 technology not only provides a molecular tool for investigating biological questions in depth, but also enables the development of innovative and practical applications of biology (Pennisi, 2013; Doudna and Charpentier, 2014; Hsu et al., 2014).

The CRISPR/Cas9 technology is a modern, fashionable method in plant research. Immediately after its early use to edit the genomes of animals and bacteria (Cong et al., 2013; Hwang et al., 2013; Jiang et al., 2013a; Mali et al., 2013b), its efficacy was demonstrated in the model plant systems of *Arabidopsis*, rice, sorghum, and tobacco (Feng et al., 2013; Jiang et al., 2013b; Li et al., 2013; Mao et al., 2013; Miao et al., 2013; Nekrasov et al., 2013; Shan et al., 2013; Xie and Yang, 2013). Nowadays, this technology is broadly used in different plant species, and dozens of CRISPR/Cas9 vectors are available in the public plasmid repository of Addgene (http://www.addgene.org/crispr/plant/). The CRISPR/Cas9 pioneers have improved this system into a flexible and powerful platform for genome engineering. In this review, we briefly summarize the recent advances of CRISPR/Cas9 technology and its impact for plant genome engineering.

## Sequence-specific DNA targeting with the CRISPR/Cas9 system

**Figure 1:**
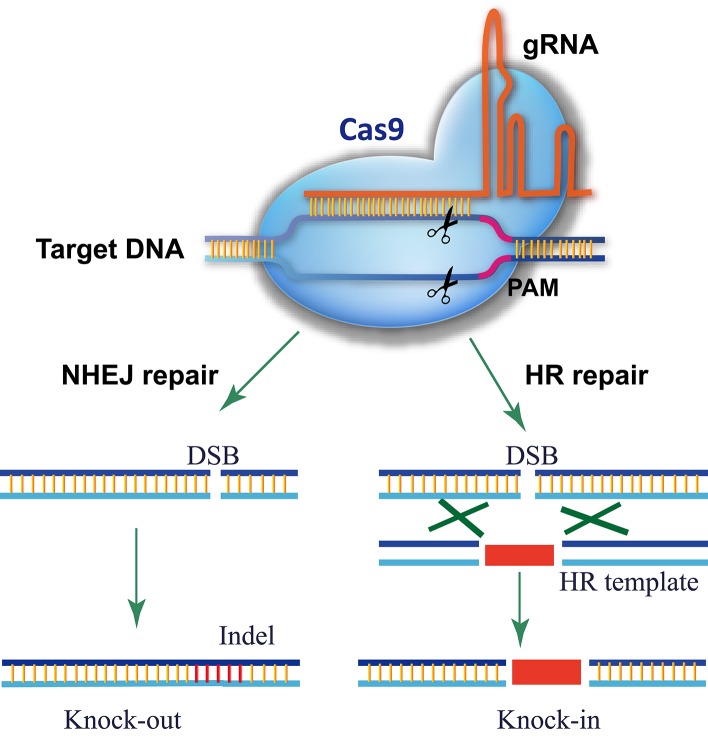
**Schematic of Cas9/gRNA genome editing**. Cas9 is directed to its DNA target by base pairing between the gRNA and DNA. A PAM motif downstream of the gRNA-binding region is required for Cas9 recognition and cleavage. Cas9/gRNA cuts both strands of the target DNA, triggering endogenous DSB repair. For a knockout experiment, the DSB is repaired via the error-prone NHEJ pathway, which introduces an inDel at the DSB site that knocks out gene function. In a knock-in experiment, the DSB is repaired by HDR using the donor template present, resulting in the donor DNA sequence integrating into the DSB site.

The CRISPR/Cas9 system comes from the adaptive immune system of bacteria and archaea, which detects and degrades invasive DNA from bacteriophages and plasmids (Fineran and Charpentier, 2012). The most commonly used RGN in genome editing is the Cas9 nuclease from the type II CRISPR/Cas9 system of *Streptococcus pyogenes* (Gasiunas et al., 2012; Jinek et al., 2012). In the prototype of the CRISPR/Cas9 system, Cas9 was directed to the DNA target by an RNA duplex of crRNA and tracrRNA, but a single guide RNA (gRNA) is used instead for genome editing (Gasiunas et al., 2012; Jinek et al., 2012, 2013; Cong et al., 2013; Mali et al., 2013b). As shown in Figure 1, there are three requirements for Cas9-mediated genome editing: (1) a Cas9 protein with a nuclear localization signal; (2) a gRNA consisting of a guide sequence (referred to as the gRNA protospacer, 20 nt) at the 5′-end that matches the DNA sequence of the target site and a conserved 3′-end scaffold with a special stem-loop structure that binds Cas9; (3) a protospacer-adjacent motif (PAM, sequence of 5′-NGG-3′) in the genomic sequence downstream of the targeted DNA. In theory, any genomic sequence bearing a PAM could be edited by Cas9 with a specific gRNA. Owing to the high occurrence of PAMs in genomes, Cas9/gRNA can target almost every gene.

Cas9-mediated plant genome editing can be easily implemented using plasmid vectors containing the Cas9 and gRNA expression cassettes. Normally, a DNA-dependent RNA polymerase II (Pol II) promoter and a Pol II transcriptional terminator are used to express Cas9 fused with a nuclear localization signal peptide, and a Pol III promoter and terminator is used to express the gRNA. The Cas9 and gRNA expression cassettes are often put in one plasmid, which is then delivered into plant cells using conventional transformation methods. Alternatively, one can microinject or transfect *in vitro*–synthesized Cas9 mRNA (or protein) and gRNA(s) into animal embryos (Kim et al., 2014; Ramakrishna et al., 2014) and plant protoplasts (Woo et al., 2015). This DNA-free genome-editing approach has sparked new breeding technologies based on CRISPR/Cas9. However, because the regeneration capacity of protoplasts is very low for most plant species, the direct injection method only suits few plants. In addition to targeted genome editing, the CRISPR/Cas9 system is also a versatile platform to manipulate genomes for different purposes.

## Targeted gene knockout using Cas9/gRNA in plants

The functional knockout of a target gene is a basic step in plant genetic analysis. In principle, it could be readily obtained using Cas9/gRNA to introduce InDels into the coding region, which will disrupt translation (Figure 1). Indeed, the Cas9/gRNA method has been successfully used to create gene knockouts in *Arabidopsis*, rice, tobacco, and sorghum (Kuhn and Binder, 2002; Feng et al., 2013; Li et al., 2013; Mao et al., 2013; Miao et al., 2013; Nekrasov et al., 2013; Shan et al., 2013; Xie and Yang, 2013). These early proof-of-concept studies proved that Cas9/gRNA generates InDels at target sites and functionally knocks out target genes with variable efficiency. Further analysis found that the edited gene faithfully passed on to the next generation of *Arabidopsis* (Feng et al., 2014) and rice (Zhang et al., 2014). In the past 3 years, CRISPR/Cas9-mediated genome editing has been established in many important crops including maize (Liang et al., 2014; Svitashev et al., 2015), wheat (Wang et al., 2014), barley (Lawrenson et al., 2015), soybean (Jacobs et al., 2015; Li et al., 2015), tomato (Brooks et al., 2014), sweet orange (Jia and Wang, 2014), petunia (Zhang et al., 2016), rapeseed (Lawrenson et al., 2015), and poplar (Fan et al., 2015; Zhou et al., 2015). The Cas9/gRNA system has become the primary choice for creating knockout mutants in plants.

In these early plant genome-editing experiments, Cas9/gRNA had a higher knockout frequency in rice than in *Arabidopsis*. Bi-allelic mutations were frequently detected in the T0 generation of genome-edited rice lines; however, most *Arabidopsis* T1 lines carried somatic mutations and required several generations to obtain the knockout mutants. For example, in *phytoene desaturase 3* (*PDS3*) knockout experiments, which evaluated knockout efficiency in plants, the albino phenotype of the *pds3*-null mutant was observed in rice (Shan et al., 2013), but not in *Arabidopsis* (Li et al., 2013; Nekrasov et al., 2013), in the primary generation of transgenic plants. It is likely that DSB repair capacity differs across recipient cell types, affecting editing efficiency and/or the timing of InDel formation during the transformation process.

This phenomenon prompted researchers to optimize Cas9 expression in *Arabidopsis* using cell- and tissue-specific promoters. In comparison to the previous constitutive 35S promoter, Cas9/gRNA editing efficiency increased after expressing Cas9 under different promoters such as the dividing cell–specific *INCURVATA2* promoter (Hyun et al., 2015), various egg cell–specific promoters (Wang et al., 2015), the cell division–specific *YAO* promoter (Yan et al., 2015), and the germ-line-specific *SPOROCYTELESS* promoter (Mao et al., 2016). These successful examples imply that the spatiotemporal expression of Cas9/gRNA is important for highly efficient genome editing in *Arabidopsis*. Because the genome editing outcome is also dependent on the endogenous DSB repair machinery, further elucidation of the DNA repair mechanism would facilitate development of more efficient genome-editing tools based on Cas9/gRNA.

The capacity of the gRNA cassette is critical for multiplex genome editing. However, the promoter used to express a single gRNA in plants has more restrictions than the Cas9 promoter. This is because the gRNA is a small non-coding RNA and requires an accurate 5′-end to keep its target-specific spacer sequence. In Cas9 genome editing, the most commonly used promoters are the snoRNA U3 and U6 genes from different plant species. To simultaneously knock out multiple genes, different gRNA expression cassettes can be inserted into one plasmid, thus guiding Cas9 to different targets (Xing et al., 2014; Zhou et al., 2014; Lowder et al., 2015; Ma et al., 2015). However, transcripts from U3 and U6 promoters are obligated to start with the nucleotides “A” and “G,” respectively. Such a restriction reduces the targeting range and potentially the efficiency of Cas9. Alternative ways to express gRNA have been developed, which enable more flexible and robust gRNA expression than did the original U3 and U6 promoters. In these, the gRNA is fused with an RNA fragment, and engineered ribonucleases excise the gRNAs from the transcript inside the cell. Three RNA processing systems have been engineered for gRNA processing: self-cleaving ribozyme (Gao and Zhao, 2014), the ribonuclease Csy4 (Nissim et al., 2014), and the endogenous transfer RNA (tRNA) processing system (Xie et al., 2015). Csy4 is an RNA endonuclease from the CRISPR/Cas system in *Pseudomonas aeruginosa* and specifically binds and cleaves a 28-nt RNA sequence (Qi et al., 2012). When this RNA sequence flanks gRNAs, Csy4 will cleave out the gRNA. After optimization to enhance the expression of this system, the Csy4 method now has efficient gRNA expression and processing for multiplex genome editing in human cells (Nissim et al., 2014), but its efficacy in plants requires further study. A strategy for robust expression of many different gRNAs in plants, as well as potentially all living organisms, is hijacking the tRNA processing system. The primary transcript of tRNA is precisely cut at both ends by endogenous RNases. Because the tRNA structure is sufficient for recognition and cleavage by these RNases, artificial genes with polycistronic tRNA-gRNA could be used for multiplex genome editing. An additional benefit is that tRNA contains an intragenic *cis*-element for Pol III transcription; thus tRNA can also be used as a transcriptional enhancer for the expression of polycistronic tRNA-gRNA gene. Indeed, the tRNA-gRNA method boosts Cas9 targeting capability (Xie et al., 2015).

**Figure 2:**
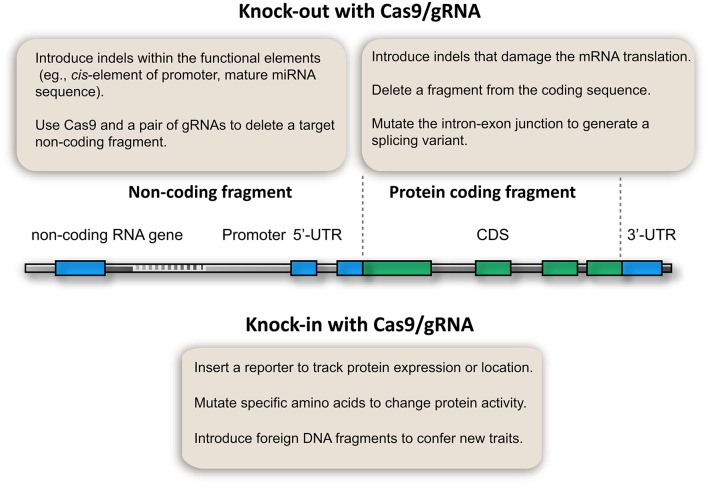
**Potential applications of CRISPR/Cas9 genome editing in plant genetic analysis**.

After 3 years of development, a fruitful CRISPR/Cas9 toolbox is available for targeted gene knockout in plants. Particularly, multiplex gRNA expression enables the development of innovative applications that benefit genetic analysis (Figure 2). This toolbox not only enables the knockout of coding genes, but also allows the study of non-coding elements, which have been a challenge in plant genetic analysis (Figure 2). For example, microRNA genes could be knocked out by introducing InDels at the mature sequence if a PAM is available, thus disturbing the function of the mature microRNA. Alternatively, short microRNA fragments could be deleted using Cas9 and multiple gRNAs. Thus, microRNA gene and other non-coding elements (e.g., *cis*-elements of promoters, enhancers, and transposons) can be knocked out (Figure 2). This proposed method is supported by the fact that the Cas9/gRNA system can generate stable plant lines with deletions of chromosomal fragments (Zhou et al., 2014; Xie et al., 2015). Owing to its simplicity and low cost, the Cas9/gRNA system can be used to generate knockout libraries covering the whole genome or a specific set of genes. Such genome-edited lines would not only give insights into plant biology, but also provide valuable genetic materials for crop breeding.

## Cas9-mediated site-directed mutagenesis and knock-in in plant genomes

Harnessing the site-directed mutagenesis and site-specific integration of a gene (a knock-in) is of great value in precision crop breeding. In this procedure, HDR repair of DSBs using a supplied DNA template results in the donor sequence (e.g., an herbicide resistance gene) being substituted into the specified region (Figure 1). Cas9/gRNA-mediated site-directed mutagenesis and knock-in has been used in rice and *Arabidopsis* protoplasts (Li et al., 2013; Shan et al., 2013). Most recently, CRISPR/Cas9 was successfully used to introduce two substitutions in an acetolactate synthase gene in rice, which confers resistance to the herbicide bispyribac sodium (Sun et al., 2016). However, generating stable knock-in plants is still a challenge for most plant species because of several technical challenges. A donor DNA template must be co-delivered into recipient cells, which increases the complexity of the experiment. Moreover, NHEJ occurs at a much higher frequency than HDR during DSB repair (Ray and Langer, 2002); thus it requires additional selection markers and more labor to identify the real knock-in lines from the other transformed plants. Among the efforts to overcome these technical challenges, a remarkable method is using the plant DNA virus replicon as the donor template (Baltes et al., 2014). The high copy number of donor DNA in geminivirus replicons greatly increases knock-in efficiency in plants. Nevertheless, CRISPR/Cas9 provides a simple method to generate a DSB at a target site to trigger HDR repair, but further optimization is still required to increase the efficiency of site-directed mutagenesis and knock-in for plant genome editing.

## Cas9-mediated transcriptional regulation of target genes

**Figure 3:**
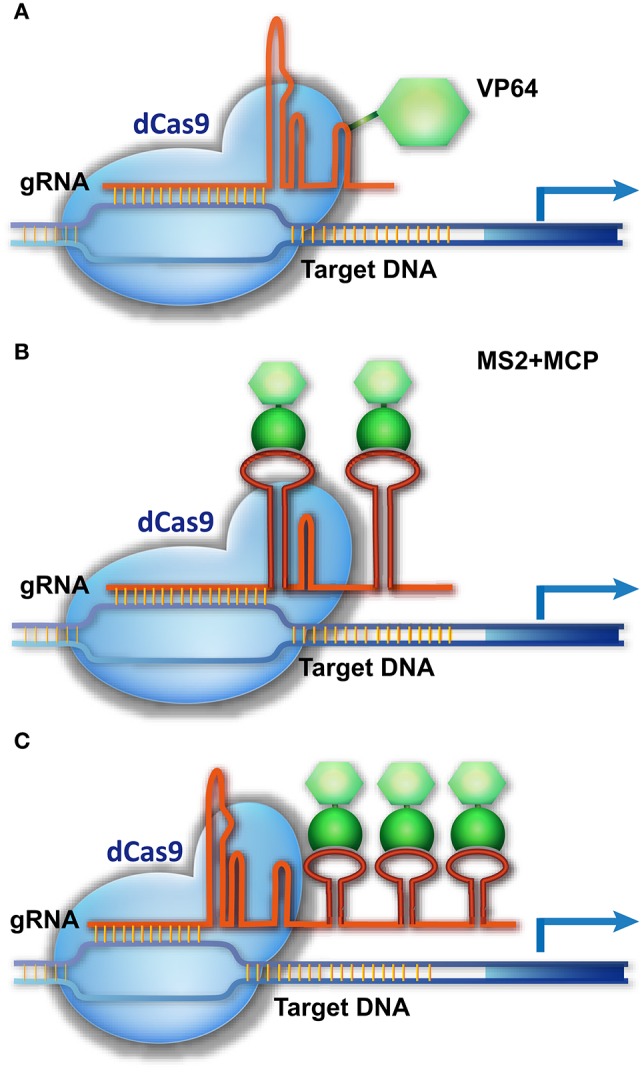
**CRISPR/Cas9-based transcriptional activation of target genes. (A)** The dCas9-AD is guided to the promoter of a target gene, activating transcription. **(B,C)** A three-component system for target gene activation. The gRNA is modified by adding an RNA ligand, MS2, to its loop or 3′-end, without impairing its binding to dCas9. MCP, which specifically binds the MS2 ligand, is fused with the AD fragment. The dCas9/gRNA-MS2 is directed to the promoter of the target gene and recruits MCP-AD to activate transcription.

Cas9 is not only a molecular scissor to edit genomic sequence, but also a versatile platform for delivering different regulatory components to a specific site. After removing the catalytic activity of Cas9 through D10A and H804A substitutions (dCas9), dCas9 and gRNA can be used as a shuttle for carrying different cargos to the DNA targets. dCas9 was engineered as a powerful tool for different purposes, particularly for transcriptional control of target genes. Early experiments in animals suggest that dCas9 fused with a transcriptional activation domain (AD, e.g., VP64) and guided by gRNA can activate the expression of the target gene (Figure 3), but 2–5 gRNAs are required to simultaneously target one promoter for robust transcriptional activation (Cheng et al., 2013; Gilbert et al., 2013; Maeder et al., 2013; Perez-Pinera et al., 2013; Chavez et al., 2015).

A more delicate strategy is using the gRNA scaffold as the platform for carrying transcriptional regulatory components to the genomic targets (Figure 3). This strategy integrates an RNA ligand and binding protein (e.g., MS2 and MCP [MS2 coat protein]) into the dCas9/gRNA system, creating a three-component system (Konermann et al., 2015; Zalatan et al., 2015). In this system, a specific RNA ligand (MS2) is added into the loop or 3′-end of the gRNA, without impairing its binding with Cas9, and the RNA ligand–binding protein (MCP) is fused with the AD domain. As a result, the dCas9/gRNA-MS2 recruits MCP-AD to its DNA target for transcriptional activation (Figure 3). In this system, multiple MS2 molecules could be added to the gRNA scaffold to simultaneously recruit several MCP-AD effector proteins for robust transcriptional activation (Figure 3). As a result, a single modified gRNA is sufficient for activating one gene (Konermann et al., 2015; Zalatan et al., 2015). The AD domain can be replaced by a transcriptional suppressor or other DNA-modifying enzyme to manipulate gene transcription (Zalatan et al., 2015) and epigenetic status (Hilton et al., 2015), respectively. Together with multiple gRNA-expressing devices, this three-component system can be used to execute complicated transcriptional programs to modulate cell activity.

In plants, target gene activation and suppression using dCas9 and gRNA was verified using the agroinfiltration transient expression system in *Nicotiana benthamiana* leaves (Piatek et al., 2015). However, similar to human cells, multiple gRNAs are required to target one promoter for robust activation or suppression. The three-component system of dCas9/gRNA-MS2/MCP-AD should enable more robust transcriptional control in plants, though additional optimization might be required. The transcriptional reprogramming capacity of dCas9/gRNA would enable us to modulate a quantitative trait and to redirect the metabolome to produce valuable nutrients or bio-agents through controlling the expression of multiple genes in plants.

## The off-target activity of Cas9/gRNA in genome editing

The off-target activity of Cas9 used to be a major concern as it can edit a DNA target bearing as many as five mismatches to its gRNA (Cradick et al., 2013; Fu et al., 2013). This off-target effect has been thoroughly analyzed by different *in vitro* and *in vivo* approaches (Hsu et al., 2013; Mali et al., 2013a; Pattanayak et al., 2013), and the fidelity of Cas9/gRNA can be summarized as follows: (1) In most cases, Cas9/gRNA cannot recognize a DNA site bearing more than three mismatches; (2) Cas9/gRNA cannot recognize and edit a DNA site with any number of mismatches near a PAM (within 10–12 bp); (3) The higher the concentration of Cas9/gRNA, the greater the potential for off-target effects; (4) Some 5′-NAG-3′ PAM sites can be targeted by Cas9/gRNA in bacteria and in *in vitro* experiments, but Cas9 has much less affinity for NAG-PAM than for NGG-PAM. Furthermore, methods based on next-generation sequencing, such as GUIDE-seq (Tsai et al., 2015), Digenome-seq (Kim et al., 2015), and ChIP-seq (Kuscu et al., 2014), can identify off-target sites for Cas9/gRNA. These high-throughput analyses also confirmed that Cas9 has off-target activity and gRNA design is essential to reduce Cas9 off-targeting risk (bioinformatic tools can help determine this, see below).

The off-target editing of Cas9 has also been observed in rice protoplasts (Xie and Yang, 2013). However, a genome-wide survey of gRNA design revealed that a sufficient number of highly specific gRNAs could be designed to cover ~90% of genes for seven out of eight plant species (Xie et al., 2014). This survey also explains why few off-target edits have been observed in plants.

Though highly specific gRNA might be designed in practice, the off-target activity of Cas9 reduces the number of its targetable sites. To overcome this pitfall, several methods have been developed to increase Cas9 targeting fidelity. Early efforts modified Cas9 into a nickase (Cas9 with either a D10A or H804A substitution and dCas9-FokI; Ran et al., 2013a; Guilinger et al., 2014; Tsai et al., 2014). These nickases need a pair of gRNAs to edit one site, thus needing 40 bp of sequence, which reduces the possibility of off-target effects. Fusion of Cas9 with additional DNA-binding domains also reduces off-target editing (Bolukbasi et al., 2015). These modifications reduced the off-target risk, but also increased the complexity. Of note, shortening the gRNA spacer sequence to 17–18 nt increases targeting fidelity (Fu et al., 2014). Recently, the off-target problem was addressed by a more elegant approach. New Cas9 variants that are intolerant to any number of mismatches have been engineered by substituting 3–4 amino acids in Cas9 (Table 1; Kleinstiver et al., 2016). These high fidelity Cas9 variants could be immediately adopted to address the off-target editing issue in plants.

**Table 1:** **Cas9 variants and orthologs for genome editing**.

**Cas9 (species)**	**PAM sequence (5′->3′)**	**References**
**Cas9 (*****STREPTOCOCCUS PYOGENES*****)**		
Cas9 wild type	NGG	Cong et al., 2013; Hwang et al., 2013; Ran et al., 2013b
Cas9 D1135E	NGG (reduced NAG binding)	Kleinstiver et al., 2015b
Cas9 37R3-2 (37R3-2 intein inserted into Cas9)	NGG (higher specificity)	Davis et al., 2015
Cas9 (N497A-R661A-Q695A-Q926A)	NGG (no detectable off-target effects)	
Cas9 VRER variant	NGCG	Kleinstiver et al., 2015b
Cas9 EQR variant	NGAG	Kleinstiver et al., 2015b
Cas9 VQR variant	NGAN or NGNG	Kleinstiver et al., 2015b
Cas9 (N497A/R661A/Q695A/Q926A), also referred to as Cas9-HF1	NGG (no detectable off-target effects)	Kleinstiver et al., 2016
Cas9 (K810A/K1003A/R1060A), also referred to as eSpCas9 (1.0)	NGG (reduces off-target effects and maintains robust on-target cleavage)	Slaymaker et al., 2016
Cas9 (K848A-K1003A-R1060A), also referred as eSpCas9 (1.1)	NGG (no detectable off-target effects)	Slaymaker et al., 2016
**Cas9 ORTHOLOGS**		
SaCas9 (*Staphylococcus aureus*)	NNGRRT or NNGRR(N)	Kleinstiver et al., 2015b; Ran et al., 2015
SaCas9 KKH variant	NNNRRT	Kleinstiver et al., 2015a
SaCas9	NAG or NGA or NNGGGT	Kleinstiver et al., 2015b; Steinert et al., 2015
FnCas9 variant (*Francisella novicida*)	YG	Hirano et al., 2016
Cpf1 (*Francisella novicida*)	TTN	Ran et al., 2015
StCas9	NNAGAA or NNGGAA	Kleinstiver et al., 2015b; Steinert et al., 2015
NmCas9 (*Neisseria meningitides*)	NNNNGATT	Hou et al., 2013; Lee et al., 2016
StCas9 (*Streptococcus thermophiles*)	NNAGAAW	Deveau et al., 2008

## Cas9 variants and orthologs for genome engineering

Besides off-target issues, the PAM requirement also restrains the Cas9 targeting range, even though the PAM sequence of 5′-NGG-3′ appears at high frequency in genomes (5–12 times in every 100 bp for model plant species; Xie et al., 2014). To bypass the PAM limitation, a number of Cas9 orthologs from type II CRISPR-Cas systems were characterized and engineered for genome editing (summarized in Table 1). These Cas9 orthologs recognize different PAM sequences and are smaller than the commonly used *S. pyogenes* Cas9, providing useful alternatives for RNA-guided genome engineering. Among these Cas9 orthologs, Cpf1 and FnCas9 recognize a PAM sequence of 5′-TTN-3′ and 5′-YG-3′, respectively (Ran et al., 2015; Hirano et al., 2016), which is present in genomes at an equal or higher frequency than 5′-NGG-3′. Because the CRISPR/Cas system is a general immunity system in bacteria and archaea, more Cas9 orthologs and other types of Cas with different PAM specificities will probably be found and engineered for genome editing in the near future.

Of note, the PAM specificities of Cas9 can be modified into other sequences by substituting several amino acid residues in the PAM-binding domain (Kleinstiver et al., 2015b). These new Cas9 variants can recognize a broad range of PAM sequences including 5′-NAGN-3′, 5′-NGCG-3′, and 5′-NGNG-3′. These improvements allow the commonly used *S. pyogenes* Cas9 to edit almost any genomic site. After adoption of these Cas9 orthologs or variants, the PAM sequence restraint will be removed.

## Bioinformatic tools for CRISPR/Cas9 applications

Many bioinformatic tools have been developed to facilitate Cas9-mediated genome editing (Table 2). These online platforms enable the design of specific gRNAs, predict the off-target sites of given gRNAs, and include other useful functions (e.g., assessing restriction enzyme cut sites; Table 2). Among these tools, only CRISPR-PLANT and CRISPR-P are specifically designed for Cas9-mediated plant genome editing. CRISPR-PLANT has a genome-wide survey of highly specific gRNAs in eight plant species and supports restriction enzyme analysis of target sites (Xie et al., 2014). CRISPR-P facilitates gRNA design for almost all plant species whose genome sequence is available and also provides off-target site analysis and restriction enzyme sequence analysis (Lei et al., 2014).

**Table 2:** **Comparison of different gRNA design tools**.

**Tools**	**Description**	**Type**	**No. of species (No. of plant species)**	**Functions**						
				**A**	**B**	**C**	**D**	**E**	**F**	**G**
Cas9 Design	A gRNA sequence design platform for the Cas9/CRISPR knockout system.	Web	10 (2)	Y	Y	N	Y	N	N	It shows RNA structure.
Cas-OFFinder and Cas-Designer	An ultrafast and versatile program that searches for potential off-target sites for CRISPR-Cas-derived RNA-guided endonucleases (RGEN).	Web and local software	73 (24)	Y	Y	Y	Y	Y	N	It provides information about out-of-frame scores for all RGEN targets.
CasOT	A genome-wide search for potential Cas9/gRNA off-target effects.	Perl	All sequenced genomes with GTF data	Y	Y	Y	N	N	N	__
CCTop	A CRISPR/Cas9 target online predictor that identifies candidate single gRNA target sites based on potential off-target effects.	Web and Python	23 (4)	Y	Y	Y	N	N	Y	__
CHOPCHOP	A web tool for selecting the optimal target sites for CRISPR-Cas9- or TALEN-directed mutagenesis.	Web	20 (1)	N	Y	Y	Y	Y	Y	It provides CHOPCHOP's algorithm for scoring and ranking potential target sites.
COD	High-throughput screening of Cas9 candidates and thorough analysis of Cas9 off-target sites.	Web	26 (2)	Y	Y	Y	N	Y	Y	__
CRISPR MultiTargeter	A web-based tool for automated searches of CRISPR gRNA targets, for finding highly similar or identical target sites in multiple transcripts, and for designing unique target gRNAs to particular transcripts.	Web	12 (3)	Y	Y	N	Y	Y	N	It shows the common and unique gRNA targets of the input sequences based on multiple sequence alignment.
CRISPR Primer Designer	A local program that has a user-friendly interface, can score for each candidate spacer, and generates the primers when using a certain plasmid.	C++/CLI software	Supports genome BLAST results of all organisms	Y	Y	N	Y	N	N	It provides plasmid design functions.
CRISPRdirect	CRISPRdirect is a web server for selecting CRISPR/Cas targets from an input sequence.	Web	228 (67)	N	Y	Y	Y	N	Y	It shows the Tm and TTTTs of the results (TTTTs need to be avoided in gRNA vectors with pol III promoters).
CRISPR-ERA	A web tool for automated genome-wide single gRNA design. CRISPR-ERA provides different search approaches.	Web	9 (0)	Y	Y	Y	Y	Y	Y	It computes an efficacy score (E) and specificity penalty score (S) for each gRNA.
CRISRP-P	A web tool for synthetic single gRNA design for plants.	Web	40 (40)	N	Y	Y	Y	Y	Y	It provides restriction enzyme cut site information.
CRISRP-PLANT	A platform to help researchers design and construct specific gRNAs for CRISPR-Cas9-mediated genome editing in eight plant species.	Web	8 (8)	N	Y	N	Y	N	N	__
E-CRISP	A software tool to design and evaluate target sites.	Web	54 (11)	Y	Y	Y	Y	Y	N	It provides variable input options dealing with different aspects of the design process.
GT-Scan	A tool for predicting the optimal target sites in a DNA sequence in relation to the reference genome of a specified organism.	Web	33 (4)	Y	Y	Y	Y	N	N	It has customized rules for identifying candidate targets and defining the specificity of each position.
MIT Optimized CRISPR Design	A web tool for selecting CRISPR guides by identifying possible off-target sites genome-wide and identifying guides with high target specificity.	Web	16 (1)	Y	Y	Y	N	Y	Y	__
Off-Spotter	A software that identifies gRNAs and returns all genomic locations that match a specific gRNA followed by a PAM sequence.	Web and local software	4 (0)	Y	Y	Y	Y	N	Y	It gives the positions that cannot tolerate a mismatch.
sgRNA Designer	An online tool for the design of gRNAs with high on-target activity for any gene of interest.	Web	Human and mouse (0)	Y	Y	Y	N	Y	N	__
sgRNAcas9	A software package developed for the fast design of CRISPR gRNA with minimal off-target effects.	Command-line software	All sequenced genomes with FASTA data	Y	Y	Y	Y	Y	N	__
ZiFiT	A tool for searching potential Cas9 single gRNA target sites in DNA sequences.	Web	Undefined	Y	Y	N	N	N	N	__

*Functions: A, Batch sequence mode; B, Guide-RNA design; C, Searches for potential off-target sites; D, GC content; E, Results scoring; F, Genome structure or annotation information; G, Characteristic functions*.

*URLs: Cas9 Design, http://cas9.cbi.pku.edu.cn/index.jsp; Cas-OFFinder and Cas-Designer, http://www.rgenome.net/; CasOT, http://eendb.zfgenetics.org/casot/index.php; CCTop, http://crispr.cos.uni-heidelberg.de/; CHOPCHOP, https://chopchop.rc.fas.harvard.edu/; COD, http://cas9.wicp.net/; CRISPR MultiTargeter, http://www.multicrispr.net/; CRISPR Primer Designer, http://www.plantsignal.cn/CRISPR/crispr_primer_designer.html; CRISPRdirect, http://crispr.dbcls.jp/; CRISPR-ERA, http://crispr-era.stanford.edu; CRISRP-P, http://cbi.hzau.edu.cn/crispr/; CRISRP-PLANT, http://www.genome.arizona.edu/crispr/; E-CRISP, http://www.e-crisp.org/E-CRISP/; GT-Scan, http://gt-scan.braembl.org.au/gt-scan/; MIT Optimized CRISPR Design, http://crispr.mit.edu/; Off-Spotter, https://cm.jefferson.edu/Off-Spotter/; sgRNA Designer, http://www.broadinstitute.org/rnai/public/analysis-tools/sgrna-design; sgRNAcas9, http://www.biootools.com/; ZiFiT, http://zifit.partners.org/ZiFiT/*.

The rapid development of CRISPR/Cas9 technologies and their broad use in plants demand that more functions be integrated into these online tools. For plant biologists who are not familiar with genome sequence analysis, a plug-and-play online program would help them design a CRISPR/Cas9 experiment. In addition, such bioinformatics platforms should incorporate gRNA design for different Cas9 proteins with variable PAM specificities, predict the on-target editing efficiency and off-target risk of gRNAs, and assist in selecting the best target sites for Cas9 from tens of candidates. Besides, many important crops are polyploidy or outcrossing species, the sequence variation between different alleles should be considered for gRNA design. Because CRISPR/Cas9 genome editing is emerging as a general tool, a platform that integrates Cas9/gRNA design tools with genome annotation data would further facilitate genome-engineering experiments in basic research and crop breeding.

## Conclusions and perspectives

Recent progress demonstrates that the CRISPR/Cas9 technology is becoming the ultimate molecular tool for genome engineering. Many of these Cas9-based technologies are being adopted in plants, and these tools will provide unprecedented insights into plant biology and enable us to improve crops with speed and accuracy through breeding. As genome-editing technology is widely used in plants, the safety of genome-edited crops is a matter of discussion in the plant community (Huang et al., 2016). Undoubtedly, the CRISPR/Cas9 technology is one of the most powerful tools in basic research and in crop genetic modification.

## Author contributions

KX, YD, and HL collected references. KX, YD, and LC wrote and revised the manuscript. All authors read and approved the final manuscript.

### Conflict of interest statement

The authors declare that the research was conducted in the absence of any commercial or financial relationships that could be construed as a potential conflict of interest.
